# Supplementary material for: Clinical Outcomes of Individuals with COVID-19 and Tuberculosis during the Pre-Vaccination Period of the Pandemic: A Systematic Review
Source: J Clin Med. 2022 Sep 26;11(19):5656. doi: 10.3390/jcm11195656 (PMC9570663; doi:10.3390/jcm11195656)
Supplement: Supplementary file 1 [file jcm-11-05656-s001.zip › jcm-1868372-supplementary.pdf]

Journal of
Clinical Medicine

MDPI

Supplementary Materials

**PubMed (NCBI)**	537 Records	((("coronavirus"[MeSH Terms] OR "Coronavirus Infections"[mesh] OR coronavirus[tiab]) AND (novel[tiab] OR wuhan[tiab] OR china[tiab] OR 2019[tiab])) OR "COVID-19"[Mesh] OR "SARS-CoV-2"[Mesh] OR "COVID-19 Testing"[Mesh] OR "COVID-19 Vaccines"[Mesh] OR covid[tiab] covid19[tiab] OR covid2019[tiab] OR 2019nCoV[tiab] OR SARS-CoV-2[tiab] OR SARSCoV2[tiab] OR SARSCoV-2[tiab] OR SARS-CoV2[tiab] OR HCov2[tiab] OR 2019ncov[tiab] OR 2019-ncov[tiab]) AND ("Tuberculosis"[Mesh] OR "Mycobacterium tuberculosis"[Mesh] OR "BCG vaccine"[mesh] OR tuberculosis[tiab] OR tubercular[tiab] OR tb[tiab] OR mdrtb[tiab] OR xdrtb[tiab] OR bcg[tiab] OR calmette*[tiab])
**Embase (Elsevier)**	897 Records	('coronavirus disease 2019'/de OR 'severe acute respiratory syndrome coronavirus 2'/de OR (coronavirus NEAR/3 (novel OR wuhan OR china OR 2019)):ab,ti,kw OR '2019-nCoV':ab,ti,kw OR 2019nCoV:ab,ti,kw OR COVID:ab,ti,kw OR COVID19:ab,ti,kw OR COVID2019:ab,ti,kw OR 'SARS-CoV-2':ab,ti,kw OR SARSCoV2:ab,ti,kw OR 'SARSCoV-2':ab,ti,kw OR 'SARS-CoV2':ab,ti,kw OR HCov2:ab,ti,kw OR '2019 ncov':ab,ti,kw OR 2019ncov:ab,ti,kw) AND ('tuberculosis'/exp OR 'Mycobacterium tuberculosis'/exp OR 'BCG vaccine'/exp OR tuberculosis:ab,ti,kw OR tb:ab,ti,kw OR mdrtb:ab,ti,kw OR xdrtb:ab,ti,kw OR bcg:ab,ti,kw OR calmette*:ab,ti,kw)
**WHO Global Literature on Coronavirus Disease**	847 Records	tuberculosis OR tb OR mdrtb OR xdrtb OR bcg
**Web of Science Core Collection (Clarivate)** Indexes=SCI-EXPANDED, SSCI, A&HCI, CPCI-S, CPCI-SSH, BKCI-S, BKCI-SSH, ESCI, CCR-EXPANDED, IC Timespan=All years	491 Records	TS=(("coronavirus" NEAR/3 ("novel" OR "wuhan" OR "china" OR "2019")) OR "2019-nCoV" OR "2019nCoV" OR "COVID" OR "COVID19" OR "COVID2019" OR "SARS-CoV-2" OR "SARSCoV2" OR "SARSCoV-2" OR "SARS-CoV2" OR "HCov2" OR "2019 ncov" OR "2019ncov") AND TS=("tuberculosis" OR "tb" OR "mdrtb" OR "xdrtb" OR "bcg" OR "calmette*")

Scheme S1. Search strategy to identify manuscripts regarding clinical outcomes of individuals with COVID-19 and tuberculosis.

Table S1. Biases and/or methodological issues of all included studies.

**Study No. (Reference No.)**	**First author (Publication date)**	**Location**	**Bias/Methodological issue**
1 [14]	Boulle (August 2020)	Western Cape, South Africa	Selection bias
2 [15]	Pillay-van Wyk (October 2020)	South Africa	Reporting bias
3 [16]	Torun (October 2020)	Turkey	Misclassification bias, Select patient cohort (healthcare workers)
4 [17]	Sun (April 2020)	Beijing, China	Selection bias
5 [18]	van der Zalm (June 2021)	Cape Town, South Africa	Selection bias, Select patient cohort (children)
6 [19]	Sy (July 2020)	Philippines	Reporting bias, Misclassification bias
7 [20]	Jassat (December 2020)	South Africa	Selection bias, Representative bias, Misclassification bias
8 [21]	Gajbhiye (February 2021)	Mumbai, India	Selection bias, Small sample size, Select patient cohort (pregnant and postpartum women)
9 [22]	Gupta (October 2020)	New Delhi, India	Selection bias, Small sample size
10 [23]	Fisman (September 2020)	Ontario, Canada	Misclassification bias, Reporting bias
11 [24]	Demkina (November 2020)	Russia	Misclassification bias, Selection bias
12 [25]	Kuwari (July 2020)	Qatar	Misclassification bias, Reporting bias
13 [26]	Meizhu Chen (April 2020)	Zhuhai, China	Selection bias
14 [27]	Lei (March 2020)	Daofu, Sichuan, China	Selection bias, Small sample size
15 [28]	Yu Chen (March 2020)	Shenyang, China	Selection bias, Small sample size
16 [29]	Hongyan Zhang (September 2020)	Wuhan, Hubei, China	Misclassification bias, Selection bias, Select patient cohort (cancer patients)
17 [30]	Huizheng Zhang (March 2020)	Chongqing, China	Misclassification bias, Selection bias, Small sample size
18 [31]	Bi (May 2020)	Shenzhen, China	Selection bias

All of them had some element of confound bias.
